# Biofilms’ Role in Planktonic Cell Proliferation

**DOI:** 10.3390/ijms141121965

**Published:** 2013-11-06

**Authors:** Elanna Bester, Gideon M. Wolfaardt, Nahid B. Aznaveh, Jesse Greener

**Affiliations:** 1 Department of Chemistry and Biology, 350 Victoria Street, Ryerson University, Toronto, ON M5B 2K3, Canada; E-Mail: elanna.bester@ryerson.ca; 2 Stellenbosch Institute for Advanced Study, Wallenberg Research Centre at Stellenbosch University, Stellenbosch 7600, South Africa; 3 Département de Chimie, 1045 Avenue de la Médicine, Université Laval, Quebec, QC G1V 0A6, Canada; E-Mails: nahid.babaei-aznaveh.1@ulaval.ca (N.B.A.); jesse.greener@chm.ulaval.ca (J.G.)

**Keywords:** biofilm, detachment, planktonic cell yield, erosion, microbial proliferation

## Abstract

The detachment of single cells from biofilms is an intrinsic part of this surface-associated mode of bacterial existence. *Pseudomonas* sp. strain CT07*gfp* biofilms, cultivated in microfluidic channels under continuous flow conditions, were subjected to a range of liquid shear stresses (9.42 mPa to 320 mPa). The number of detached planktonic cells was quantified from the effluent at 24-h intervals, while average biofilm thickness and biofilm surface area were determined by confocal laser scanning microscopy and image analysis. Biofilm accumulation proceeded at the highest applied shear stress, while similar rates of planktonic cell detachment was maintained for biofilms of the same age subjected to the range of average shear rates. The conventional view of liquid-mediated shear leading to the passive erosion of single cells from the biofilm surface, disregards the active contribution of attached cell metabolism and growth to the observed detachment rates. As a complement to the conventional conceptual biofilm models, the existence of a biofilm surface-associated zone of planktonic cell proliferation is proposed to highlight the need to expand the traditional perception of biofilms as promoting microbial survival, to include the potential of biofilms to contribute to microbial proliferation.

## 1. Introduction

The detachment of single cells or aggregates of cells from biofilms is increasingly recognized as an intrinsic part of this surface-associated mode of bacterial existence. The detachment or dispersal of cells from the biofilm is typically viewed as the “final” step in biofilm development, as reviewed by [1], which enables a fraction of the biofilm-associated cells to escape unfavorable conditions within the biofilm and initiate the establishment of a new biofilm elsewhere. While this conceptual view of biofilm development as a linear process, consisting of five consecutive phases [2], can be beneficial in elucidating the various biotic and abiotic factors that influence biofilm formation, it can also obscure the fact that detachment does occur during earlier stages of the biofilm life-cycle and is not restricted to “mature” biofilm microcolonies only. For example it was estimated that up to 44% of *Pseudomonas aeruginosa* PA01 cells emigrated within the first 35 h of attachment [3].

Different detachment mechanisms have been identified and are generally classified as either passive or active based on whether the removal of attached biomass is due to factors such as human intervention (*i.e*., antimicrobial treatment), the result of abiotic forces (*i.e*., increase shear or particle abrasion), or an active microbial response to environmental cues that requires genetic regulation.

The rapid dissolution of biofilms of various Pseudomonad species in response to changes in carbon and nitrogen availability has been reported [4–6]. Large numbers of planktonic cells were observed to be swimming away from a previously stable *P. putida* biofilm within minutes of the removal of carbon from the growth medium, or a cessation in the liquid flow [5]. A decrease in the amount of intracellular secondary signaling molecule cyclic-di-GMP was found to induce biofilm dispersal through the induction of the LapG cysteine proteinase, which in turn modified the adhesion protein LapA, thereby dissolving the biofilm [7]. This observation indicates that individual cells have the ability to dissociate themselves from their neighbors and/or the biofilm matrix.

“Seeding dispersal”, describes a process where large (>80 μm diameter) *P. aeruginosa* PAO1 microcolonies break open to release a subpopulation of planktonic cells into the bulk-liquid [8]. It is as yet unclear how widespread this phenomenon is among different bacterial species or whether the above-mentioned global dispersion-response to changes in the environment occurs by the same mechanism(s).

From a mass balance point of view, biofilm development is the net result of biomass accumulation and biomass detachment or decay. Indeed, biofilm biomass does not accumulate indefinitely, but instead a *pseudo* steady state is reached, where further growth is balanced by detachment or decay. Passive biofilm detachment mechanisms have been defined, somewhat arbitrarily, as erosion or sloughing, based on the detachment frequency and size of the detached particle, in addition to biomass removal due to abrasion, predator grazing or human intervention strategies [9]. The term “erosion” is frequently used to describe one of the most common, and likely underestimated, biofilm detachment processes. While erosion is defined in the Merriam-Webster Learner’s Dictionary as “the gradual destruction of something by natural forces”, or “the process by which something is worn away”, in biofilm-related literature the term “erosion” has been defined as the “continuous removal of small particles of biofilm” and is “presumed to be the result of shear forces exerted by moving fluid in contact with the biofilm surface” [10]. In order to sustain a continuous detachment rate while maintaining a *pseudo* steady state biofilm structure, continuous growth by the attached biomass is required. In fact, from a theoretical examination of a biofilm mass balance, it is evident that the maximum sustainable detachment rate is governed by the biomass growth rate [10].

Previous work by Rittman in 1982 [11] proposed a model wherein the rate of biofilm erosion due to liquid shear stress was dependent on the amount of attached biofilm, based on the analysis of a subset of empirical data produced by Trulear and Characklis (1982) [12] for aerobic, multispecies biofilms cultivated in an annular reactor and subjected to different shear forces. Subsequent experimentation by Peyton and Characklis (1993) [10] investigated the response of multispecies and pure culture *Pseudomonas aeruginosa* biofilms to variations in shear stress (1.44, 2.20, or 2.97 Pa) and substrate availability (0.8, 4.0, or 7.2 grams glucose carbon m^−3^). Biofilm substrate utilization rates and erosion rates were shown to increase with an increase in biofilm biomass, as proposed by the previous model [11]. However, contrary to this model, the erosion rates were not directly related to the applied liquid shear stress, but instead to the substrate loading rates; where an increase in carbon availability led to an increase in the detachment rate. The authors furthermore re-analyzed the full data set previously published [12] and found no indication of a linear relationship between biofilm erosion rates and the applied shear stress (*r*^2^ = 0.00038).

Stewart (1993) [13] further reinforced the link between a metabolically active biofilm region, responsible for carbon utilization, and biofilm detachment. Several mathematical expressions were derived to explain biofilm detachment, one of which explicitly accounted for spatial variation of growth rates within the biofilm. This expression provided a good qualitative prediction of detachment rates for the empirical steady state data from Trulear and Characklis (1982) [12].

Despite the existence of these publications, the presence of single cells in the effluent of continuous-flow biofilm cultivation systems is still often ascribed to the erosion of the biofilm surface solely due to liquid shear stress [14–16].

Previous work by our group has detailed the extent to which single and multi-species biofilms cultivated under continuous flow conditions in glass tubes produced and released planktonic cells to the bulk-liquid. Contrary to the accepted 5-stage model of biofilm development where dispersion of single cells from the biofilm only takes place upon maturation of a microcolony, an increase in planktonic cell numbers was evident as early as 6 hours after reactor inoculation [17,18]. The planktonic cell numbers in the effluent continued to increase during biofilm development and generally reached a plateau after 3 to 4 days, likely once the biofilm biomass reached a *pseudo* steady state. Variation in the bulk-liquid flow rates, leading to average shear rates ranging from 19.1 × 10^−3^ mPa to 93.9 mPa, did not result in a linear response in the magnitude of planktonic cells enumerated from the effluent, as would be expected if shear erosion was the dominant determinant of detachment (use of the term “average” to describe shear rates is due to the variation in shear rates along the cross section of a channel; lower shear rates will be present near the walls, with high shear rates in the center). Instead, the impact of reduced flow rates on the availability of nutrients and/or oxygen on biofilm development was found to be a greater determinant of planktonic cell numbers [18]. The removal of the sole carbon source, while maintaining a constant average shear stress, was shown to not only reduce the metabolic activity of a biofilm to below detection limits, but was accompanied by a 1 to 2 order of magnitude reduction in the number of viable planktonic cells produced by the biofilm [19]. Upon the re-introduction of carbon after eight days of starvation, the pre-disturbance levels of planktonic cell yield and CO_2_ respiration rates were re-established within 24 h.

To distinguish these observations from the widely held erosion-mediated cell removal and to emphasize the role played by microbial physiology, we proposed the use of the term “biofilm-derived planktonic cell yield” to describe this process.

The use of square glass tubes with a comparatively large cross sectional area (0.04 cm^2^), restricted the period of time during which biofilms could be subjected to high shear rates due to the large volumes of sterile growth media required. For example, a continuous flow rate of 450 mL h^−1^, which results in an average shear stress of 93.9 mPa in an uncolonized glass tube, could only be maintained for 12 h in a typical laboratory continuous flow system [18]. Using microfluidic flowcells, we quantify various biofilm parameters as well as biofilm-derived planktonic cell yield under higher average shear stresses than those applied previously to determine whether *Pseudomonas* sp. strain CT07*gfp* biofilms can maintain their physical structure (biomass) as well as the continuous yield of planktonic cells if subjected to significant removal forces.

## 2. Results and Discussion

### 2.1. Biofilm-Associated Planktonic Cell Production

The bulk-liquid flow rates at which the growth medium was supplied to the microfluidic channels resulted in dilution rates that greatly exceeded the maximum rate at which *Pseudomonas* sp. strain CT07*gfp* can replicate when growing in suspension. Consequently, it is assumed that an independently replicating planktonic population of bacteria would be incapable of persisting in the bulk-liquid phase of the channels. The culturable cell numbers enumerated from the effluent samples were thus presumed to originate from surface-associated growth. The majority of the detached biomass particles in the effluent consisted of single or dividing cells, whereas the presence of large sloughed aggregates of cells were rarely observed (data not shown).

**Figure 1:**
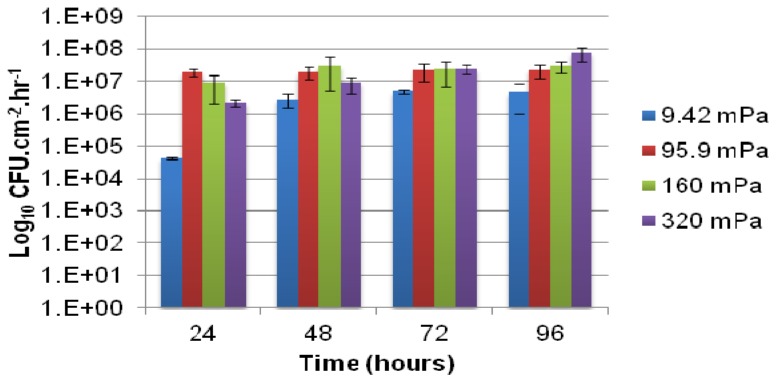
The viable cell numbers (colony forming units (CFU) per mL of effluent) released from biofilms, normalized with respect to the bulk-liquid flow rate (mL h^−1^) and total substratum area (cm^2^) available for cell attachment and biofilm development. Biofilms were allowed to develop in replicate microfluidic channels for up to 96 h under different bulk-liquid flow velocities; thereby subjecting the biofilms to a wide range of average fluid shear rates (mPa). Error bars indicate the standard deviation of samples taken from replicate biofilms (two experimental rounds, with each round consisting of biofilms growing in two microfluidic channels).

Comparatively small differences in the rate of planktonic cell production by biofilms subjected to a large range of shear stresses is evident from Figure 1, apart from the lowest shear rate (9.42 mPa) where the numbers of cells present in the effluent were consistently lower. Statistical analysis indicated a significant difference between the cell yield from biofilms subjected to the lowest shear stress (9.42 mPa) and those at higher shear stresses (*P*_0.05_ = 1.36 × 10^−5^). The reduced flux of nutrients to the biofilm was probably a major cause for the lower cell yield, which is in agreement with the earlier observations [13] regarding the link between carbon utilization and biofilm detachment. The rate of planktonic cell production generally increased slightly over the course of incubation and peaked at the production of ~10^7^ cells per cm^2^ of internal channel surface area per hour, for biofilms subjected to the higher shear rates (95.9 mPa, 160 mPa and 320 mPa). Two-factor ANOVA and subsequent Post Hoc analysis indicated a statistically significant higher yield from 96 hour-old biofilms subjected to 320 mPa than younger biofilms exposed to the same shear stress (*P*_0.05_ = 1.14 × 10^−5^). This observation is somewhat unexpected, since the cell yield generally exhibits only minor fluctuation once biofilm development reaches a steady state after 3 to 4 days of cultivation.

### 2.2. Biofilm Morphology

**Figure 2:**
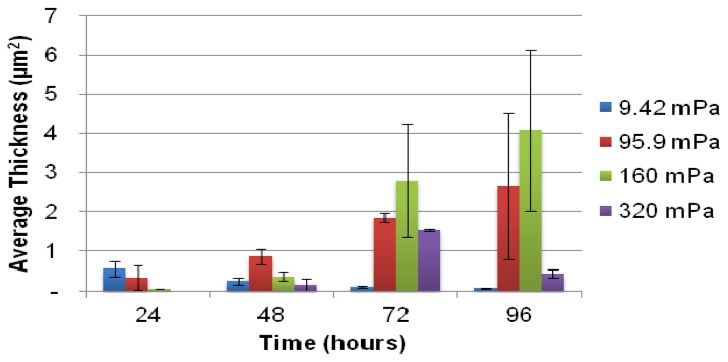
The average thickness of microfluidic channel biofilms as determined by COMSTAT analysis of replicate confocal scanning laser microscopy (CLSM) Z-stacks. The biofilms were subjected to four different bulk-liquid flow velocities to exert a wide range of bulk shear stresses on the biofilms during development. Error bars indicate the standard deviation of the average thickness of duplicate biofilms, cultivated in separate microfluidic channels. Ten microscope fields, each with an area of 101,761 μm^2^ (318 μm × 318 μm), were chosen at random along a central transect starting from the channel inlet, and a Z-stack of images was captured in the *z*-direction at 0.60 μm intervals.

**Figure 3:**
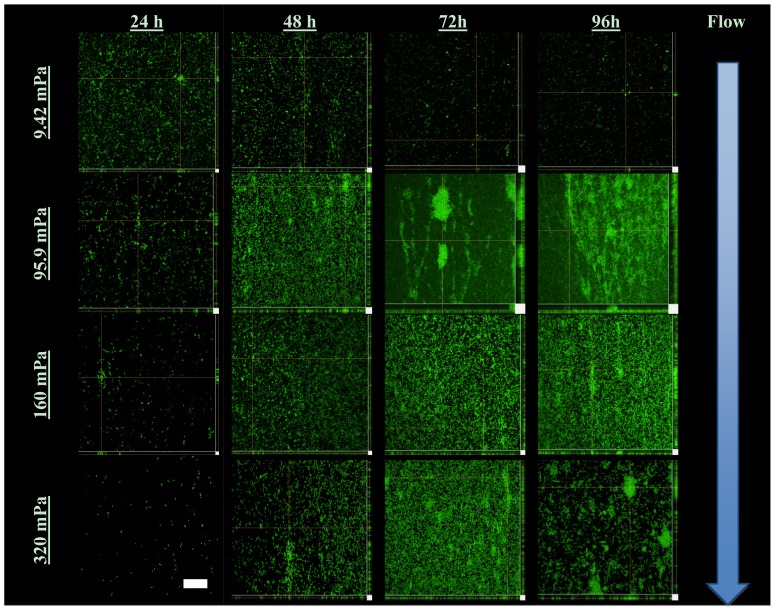
Representative CLSM orthogonal projections of stacks of images taken of the various biofilms at 0.6 μm depth intervals. The main part of each image consists of a single 2D slice of the biofilm (*xy*-direction) whereas the smaller side panels below (*xz-*direction) and to the right (*yz-*direction) indicate a digital projection of the depth of biofilm biomass from the attachment surface to the bulk-liquid interface. A scale bar indicating 50 μm is included in the bottom left image.

The extent of biofilm development was quantified by confocal scanning laser microscopy (CLSM) and subsequent analysis of average biofilm thickness with COMSTAT (Figure 2). Biofilm development under the lowest average shear rate (9.42 mPa) was significantly lower, with a decline in the amount of attached biomass over the course of 96 h (Figure 3), likely due to a lack of nutrients and/or oxygen. In contrast, the average thickness of the biofilms exposed to higher shear rates (and thus higher substrate loading rates) generally increased due to attached cell growth.

Higher flow velocities increase substrate supply and thus availability, which supports the accumulation of greater amounts of attached biomass. However, higher flow velocities also exert more significant shear stresses on the biomass, leading to a greater probability of biomass detachment. The highest shear rates applied in this study were not sufficient to prevent biofilms from developing at the glass surface, although a decrease in the average biofilm thickness at 96 h (Figure 2) may suggest that the narrowing of the channel’s cross section due to biofilm development on the channel walls increased the bulk average shear rate to such an extent that a portion of the attached biomass was unable to resist shear mediated detachment. Despite the reduction in average biofilm thickness, the yield of planktonic cells to the bulk-liquid was observed to increase, (Figure 1), which suggests that biofilms maintain their proliferation function under a wide range of flow conditions and biofilm form.

The development of chains of cells and the formation of large ridges of matrix-embedded cells interconnecting individual microcolonies were also observed. As could be expected, these ridges or filaments were oriented parallel to the direction of flow, as could be noted for 72 and 96 hour-old biofilms subjected to a shear stress of 95.9 mPa and 96 hour-old biofilms subjected to 160 mPa (Figure 3).

CLSM projections (Figure 3) illustrate the morphology of biofilms developing under the different shear stresses. The use of the 40× objective during microscopy facilitated the capture of the variation in biofilm morphology along the majority of the entire cross section of each channel. The reduction in local shear rates in the vicinity of the corners of the channels (where the glass substratum meets the walls of the microfluidic channels), compared to the higher shear removal forces operating along the center of the channel, allowed more biofilm biomass to accumulate near the wall. This is evident from the large amounts of green fluorescent biomass present towards the right hand of some of the projections (95.9 mPa at 72 and 96 h, Figure 3). The heterogeneity in the amount of biomass developing along the cross section (perpendicular to flow), as well as the length of each channel (in line with the flow from in- to outlet), is reflected in the large standard deviations in the average biofilm thickness (Figure 2).

### 2.3. Relationship between Average Shear Rates and Planktonic Cell Production Rates

**Figure 4:**
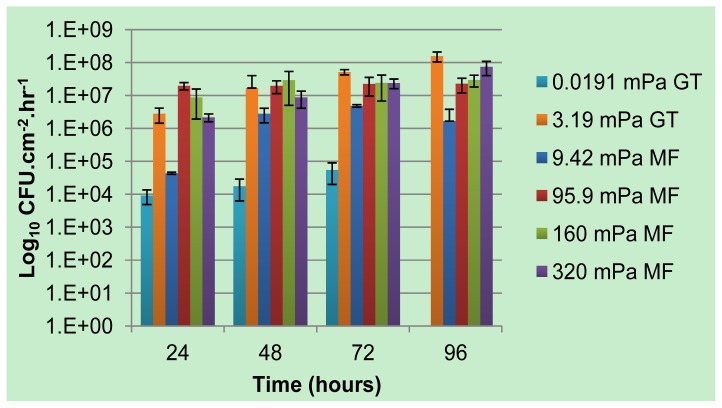
A comparison of biofilm-associated planktonic cell production rates from biofilms grown in microfluidic channels (MF, this study) and square glass tubes (GT, reference [18]) subjected to a wide range of average shear rates by varying the bulk-liquid flow rates. The inner dimensions of the square glass tubes were 2 mm × 2 mm × 152.4 mm (Friedrich & Dimmock, Inc., Millville, NJ, USA).

Previously published data [18] on the influence of shear rates on *Pseudomonas* sp. strain CT07 biofilms cultivated in macroscopic square glass tubes using the same growth medium, is combined with the data from Figure 1 and presented in Figure 4 for comparative purposes. Apart from the reduced planktonic cell production rates from biofilms subjected to the lowest average shear rates (19.1 × 10^−3^ mPa in the Glass tubes, and 9.42 mPa in the Microfluidic channels), likely due to restricted nutrient and oxygen availability, the general rates at which planktonic cells were produced and released did not vary substantially for a range of average shear stresses spanning 2 orders of magnitude. These data do not support the prevailing assumption that the magnitude of the shear stresses applied to biofilms determines the extent of single cell detachment due to erosive action.

A recent report [16] described the effect of successive step increases in wall shear stress on multispecies biofilm detachment rates; the area of the detached particles were used to empirically distinguish between eroded particles (0.04 μm^2^ to 5 μm^2^) and sloughed aggregates (>5 μm^2^). The first increase from 5.1 mPa to 21.8 mPa (4-fold increase) did not result in a significant increase in the frequency of erosion or sloughing, whereas a further doubling of shear stress to 43.6 mPa significantly increased the occurrence of sloughing as well as erosion. This led the authors to suggest that “a critical shear stress exists, below which the erosion rate is independent of shear stress”. If this statement is applicable, the highest shear stress applied in this study (320 mPa) still remains below the ‘critical shear stress’ required to erode the biofilm.

When shear mediated detachment from biofilms is modeled mathematically, the mathematical expression for the detachment rate is often defined to be dependent on biofilm thickness and/or biofilm biomass, since thicker biofilms extending into the bulk-liquid would be subjected to higher shear forces [11,20,21]. However, upon analysis of an extensive set of data [13], it was concluded that expressions of a first- or second-order dependence of the detachment rate on biofilm biomass alone did not fit the empirical data. Instead, an expression also incorporating microbial growth rates, which are dependent on substrate availability, was found to provide a better description [13].

A positive correlation between an increase in the surface area of the biofilm and the number of planktonic cells present in the bulk-liquid phase associated with biofilm reactors could support a role for both active and passive detachment mechanisms. It can be hypothesized that an increase in the biofilm surface area (due to a more varied topography) would expose a greater number of attached cells to optimal nutrient and/oxygen supply from the bulk-liquid as well as the removal of toxic metabolites. These favorable conditions would lead to higher overall growth and cell division rates and together with the increase in biofilm surface area, would increase the potential for active release of newly formed daughter cells to the bulk-liquid after cell division. Similarly, it can be argued that liquid-mediated shear erosion may also lead to the removal of more cells by passive erosive detachment from the biofilm surface upon an increase in the area of the biofilm exposed to liquid shear.

**Figure 5:**
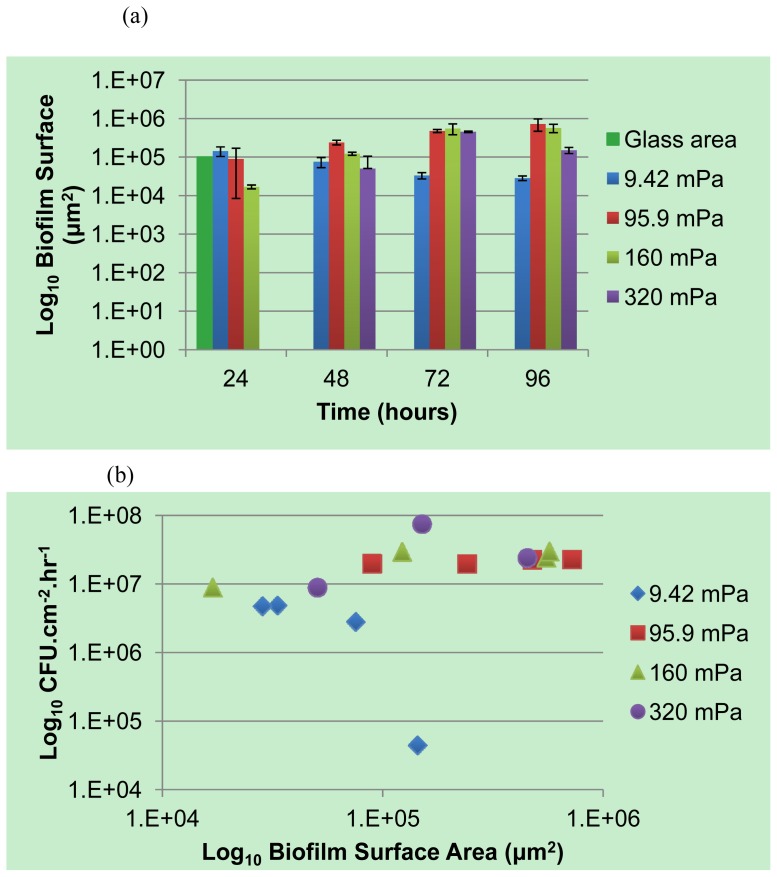
(**a**) The area of the biofilm (μm^2^) exposed to the bulk-liquid environment was calculated for each Z-stack from data obtained after COMSTAT analysis of the CLSM images. The grey bar indicates the area of the glass substratum; note that the incomplete coverage of the glass surface, and the patchy nature of biofilms as evident in Figure 3, explains why the area of the glass surface often exceeds that of the biofilms; (**b**) The relationship between the average cell production rate (Log_10_ CFU.cm^−2^ h^−1^) and the surface area of the biofilm (Log_10_ μm^2^) exposed to the bulk-liquid, for biofilms developed at 4 different average shear rates.

The biofilm surface area (μm^2^) exposed to the bulk-liquid was accordingly calculated (Figure 5a) using the results of COMSTAT analysis, as detailed in the Experimental section. To our knowledge this has not been attempted before, however, the values obtained correlated well with the degree of biofilm development, as evident from the average biofilm thickness (Figure 2) and the extent of substratum coverage (Figure 3). The values were utilized to investigate whether a correlation between the extent of planktonic cell yield and biofilm surface area could be identified for biofilms subjected to different shear forces (Figure 5b). The data from Figure 5b does not seem to support a linear relationship between the biofilm surface area and the number of cells yielded or eroded from the biofilm, for any of the different average shear rates.

This result demonstrates the self-regulating ability of biofilms to maintain function under various conditions; in this case the proliferation function, expressed as biofilm-to-planktonic cell yield, remained relatively stable when the variable was shear rate. The great variety of biofilm composition (cells and EPS matrix) and structure described in the literature suggests that a notable degree of plasticity enables such self-regulation and the ability to maintain biofilm function under varying conditions of shear stress or substrate/nutrient type and availability, reactor configuration, *etc*. It is evident from the results presented here that shear stress (*i.e*., “erosion”) alone cannot account for the numbers of biofilm-derived planktonic cells present in the effluent.

**Figure 6:**
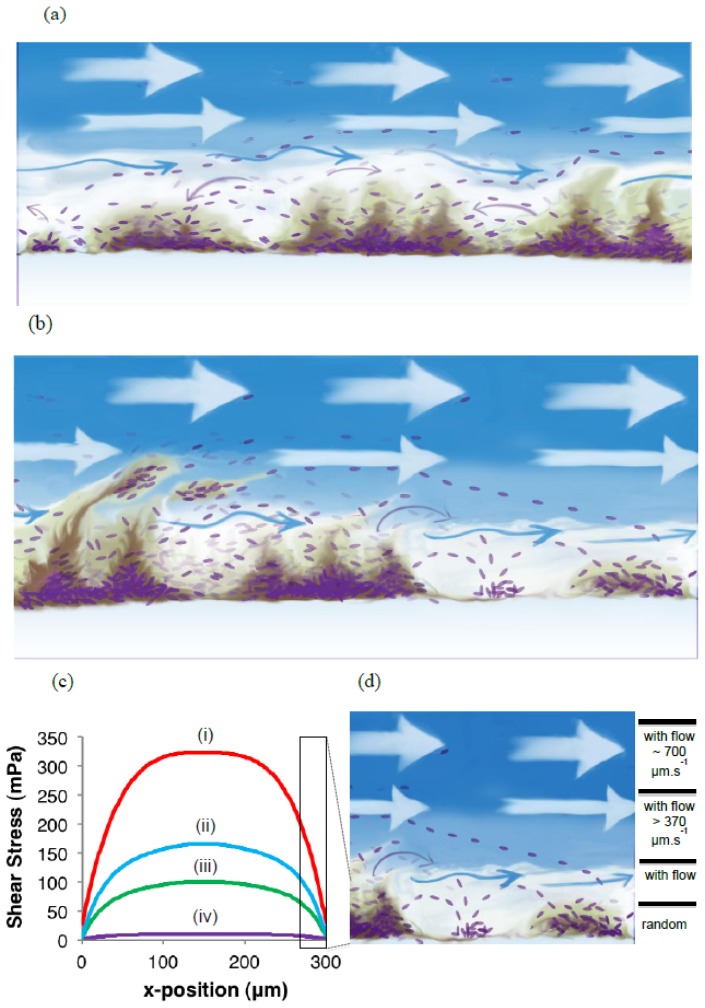
An illustration of the development of the proposed biofilm surface-associated zone of planktonic cell replication. (**a**) In the early stages of biofilm development, after individual cells colonize the surface and initiate microcolony formation, planktonic cells are produced and released; (**b**) As the biofilm continues to develop planktonic cell yield increases, despite EPS accumulation, as well as the potential for the sloughing of larger aggregates of cells embedded within EPS; (**c**) A Comsol simulation of the wall shear stress resulting from flow rates (mL h^−1^) of (i) 97.0 × 10^−2^; (ii) 48.5 × 10^−2^; (iii) 29.1 × 10^−2^; and (iv) 28.6 × 10^−3^ flowing through a microchannel with dimensions of *h* = 130 μm and *w* = 300 μm. Shear stress values approached 0 in the corners where the glass substratum meets the channel wall (at *x* = 0 μm and *x* = 300 μm) for all flow rates; (**d**) Cell movement in surface-associated zone. Single cell translocation (shown at the right of the illustration), both with respect to distance and direction, was determined by time-lapse microscopy at various distances (*z*-direction) from the solid substratum. From these observations it is evident that the outer regions of the biofilm EPS have different viscosity than that of the biofilm core. Images are not drawn to scale.

The detachment of cells from biofilms is often ascribed to the need for cells to escape unfavorable environmental conditions and to facilitate the colonization of new habitats. The potential role of biofilms, or perhaps biofilm surface-associated zones, as niches for planktonic cell production has often been overlooked. The observation that planktonic cells are able to remain associated with the biofilm [22] for extended periods indicates that their reactor residence time could be prolonged to such an extent that an independently replicating planktonic population can become established in the reactor. An illustration of such biofilm or surface-associated ‘zone of planktonic cell replication’ is given in Figure 6.

Biofilms are mostly studied using cell-specific stains, or cells tagged with fluorescent proteins, as in the current study. This, together with the highly diffused nature of EPS, complicates efforts to determine the extent of the extracellular matrix of biofilms. Coverage of biofilm microcolonies by a strongly cohesive matrix material at the biofilm bulk-liquid interface will hinder the release of cells from the biofilm (*i.e*., the concept of irreversible attachment). Considering the high yield of cells from biofilms as reported here, it is clear that such a zone of planktonic cell replication exists. In fact, the presence and close association of motile single cells with immobile, matrix-embedded biofilm cells can be shown with relative ease during microscopic investigation of biofilms under continuous flow conditions. These typically reveal that in the immediate vicinity of the biofilm, motile planktonic cells can propel themselves in any direction to interact with other microcolonies, or re-attach in an uncolonized area to initiate biofilm formation, but if they stray too close to the edge of the region of reduced flow velocity, the cells become entrained by the bulk-liquid flow (Figure 6a,b).

To illustrate the differences in flow rate, we used Comsol simulations to show the wall shear stress resulting from the four different flow rates (mL h^−1^) of (i) 97.0 × 10^−2^; (ii) 48.5 × 10^−2^; (iii) 29.1 × 10^−2^; and (iv) 28.6 × 10^−3^ flowing through a microchannel with dimensions of *h* = 130 μm and *w* = 300 μm. As shown in Figure 6c, shear stress values approach 0 in the corners where the glass substratum meets the channel wall (at *x* = 0 μm and *x* = 300 μm) for all flow rates. The maximum and average wall shear stress values (mPa) for flow rates (i–iv) were: (i) 415 mPa and 321 mPa; (ii) 209 mPa and 162 mPa; (iii) 125 mPa and 97.4 mPa; and (iv) 12.4 mPa and 9.62 mPa.

To compare microbial behavior with these flow conditions, time-lapse microscopy was used to track the movement of single cells at various vertical distances (*z*-direction) from a biofilm-colonized glass substratum. Single, planktonic cells were able to move at angles diagonal to the prevailing flow direction only while they remained within a depth similar to the average thickness of the biofilm (3.5 μm in this case) where the bulk-liquid flow velocities were sufficiently reduced. Measurements taken at greater distances from the glass substratum (up to 12 μm) indicated that while the trajectory of single cells was exclusively parallel to the direction of bulk-liquid flow, the cells travelled at reduced velocities (maximum measured 370 ± 100 μm s^−1^) compared to the bulk-liquid velocity of 700 μm s^−1^ (0.1 mL h^−1^) (Figure 6d). Particles could not be tracked at greater distances from the surface due to the high velocities, but large numbers of single cells were routinely swept past the field of view in the bulk-liquid phase. While these cells were unable to overcome the prevailing flow velocity, it can reasonably be assumed that a reduction in flow velocity or change in flow patterns (*i.e*., eddies) may carry the cells to regions of slower flow where flagellar-driven motility could propel the cells towards a surface.

The association and persistence of planktonic cells in this region of reduced flow could lead to the establishment of an independent planktonic cell population in a reactor, where nutrient availability, the accumulation of waste products and/or signaling molecules within this region of reduced flow have the potential to govern the degree of planktonic cell replication. A previous report suggested that the co-existence of motile single cells with attached cells could afford the biofilm greater flexibility to respond to environmental cues [22]. The phenotype of biofilm-derived planktonic cells has been shown to be different from planktonic cells cultivated in batch suspension. Planktonic cells yielded from *Staphylococcus aureus* biofilms exhibited reduced production of a collagen adhesin and thus a lower subsequent rate of adhesion and biofilm formation [23]. A shortened lag in *Pseudomonas* sp. strain CT07 biofilm development has been reported by employing biofilm-derived cells as inocula, as compared to cells cultivated in batch suspensions [24]. Together with the enhanced antimicrobial susceptibility previously reported [17], the existence of a third phenotype for biofilm-derived planktonic cells has been postulated [25].

The role of biofilms in planktonic cell proliferation has not been adequately recognized to date. The metabolic capabilities of self-immobilized microbial communities are exploited in wastewater treatment facilities and in the production of high value metabolites, whereas the recalcitrance of undesirable and antimicrobial resistant fouling biofilms is often lamented. However, the results presented here and elsewhere [17,18,26] indicate that these are not the only properties of biofilms with the potential to impact society. The incorporation of pathogens into environmental biofilms associated with plumbing material or medical equipment and subsequent dissemination to susceptible individuals has been documented to contribute to healthcare-associated infections and subsequent mortality [27–30]. The detachment of single cells from biofilms that develop on implanted medical devices could also lead to secondary infections at other sites, or septicemia [23].

## 3. Experimental Section

### 3.1. Strain and Culture Conditions

An environmental pseudomonad, isolated from a cooling tower and designated as *Pseudomonas* sp. strain CT07 (GenBank Accession number DQ 777633) was used for all of the experimentation [17]. A gene sequence encoding for the constitutive expression of a green fluorescent protein (GFP) was inserted into the bacterial chromosome using a mini-Tn7 system as previously described [18].

Cultivation took place at room temperature (24 ± 2 °C) in modified AB defined medium (final concentration of 1.51 mmol/L (NH_4_)_2_SO_4_, 3.37 mmol/L Na_2_HPO_4_, 2.20 mmol/L KH_2_PO_4_, 179 mmol/L NaCl, 0.1 mmol/L MgCl_2_·6H_2_O, 0.01 mmol/L CaCl_2_·2H_2_O and 0.001 mmol/L FeCl_3_) [31] with 1 mmol/L Na-Citrate·6H_2_O as the sole carbon source. Pre-cultures were incubated in batch with aeration for 16–18 h prior to the inoculation of microfluidic channels.

### 3.2. Microfluidic Device Fabrication

**Figure 7:**
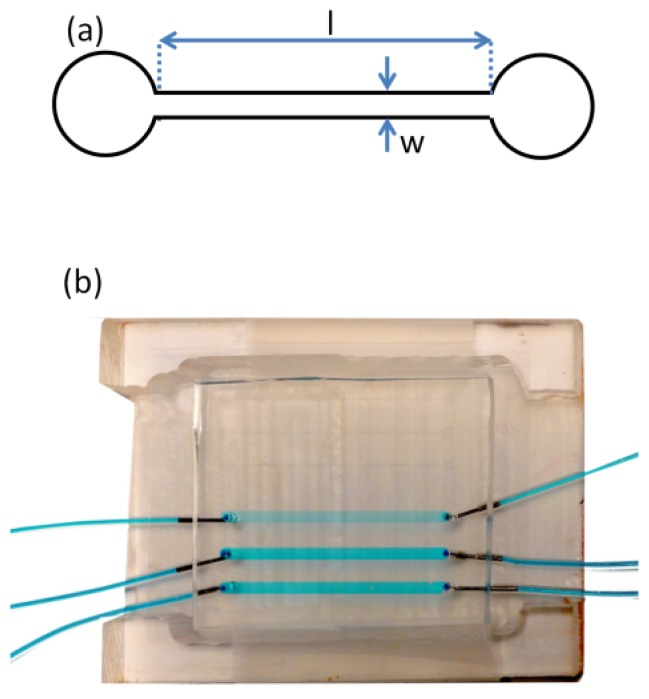
(**a**) Two-dimensional schematic for a single channel used in this study consisting of a channel width (*w*) of 300 μm and length (*l*) of 40 mm. The height (not shown) is 130 μm. The two circular sections at the end of the channel mark the inlet and outlet; (**b**) A photograph of the microfluidic device in a polycarbonate holder. The holder positions the device with the glass side up for inspection by the confocal system and provides space on the bottom (PDMS) side for liquid delivery tubing access to the inlet and outlet holes. The *w* = 2 mm channels are connected to liquid delivery tubing and filled with a blue dye for visualization.

A silicon template containing microfeatures from a photoresist (SU-8 50, Microchem Inc., Newton, MA, USA) was prepared via photolithography, based on a photo mask that was produced using computer aided design software (AutoCAD). The height of all features on the silicon template was 130 μm as defined by the spin coating process. After photolithography, the silicon template contained the inverse features required for the microfluidic device. These consisted of two sets of channels in triplicate, for statistical purposes. The first set of channels had a width (*w*) of 2 mm and the second had a width of 300 μm. All 6 channels had a length (*l*) of 40 mm. In this study, only the 300 μm-wide channels were used in order to achieve the desired range of average shear rates. Figure 7a shows a schematic of one *w* = 300 μm channel. The microfluidic devices were prepared by casting uncured poly dimethyl siloxane (PDMS) (Ellsworth Adhesives Canada, Syligard 184) against the silicon template and heating to 70 °C overnight [32]. After the cured PDMS was demoulded from the silicon template the micro fabrication contained the features of the microfluidic channel and inlet and outlet access holes were punched at each end of the channel. Finally, a glass cover slip (VWR, catalogue number CA48404-143) was bonded to the PDMS after exposure to air plasma (HARRICK PLASMA-PCD-001) for 90 s, thereby sealing the device. The coverslip thickness was 170 μm, which matched working distance requirements for the confocal microscope objectives. Liquid delivery tubing was connected to the device inlets and outlets via metal elbow capillaries and glued in place using multi-purpose silicone sealant. The entire device was placed in a custom polycarbonate holder, which positioned the device with the glass side up for inspection by the upright confocal imaging system (Figure 7).

### 3.3. Continuous-Flow Cultivation of Biofilms

Biofilms were cultivated in microfluidic channels for up to 4 days. Thick-walled silicone tubing (Cole Parmer, 0.89 mm inner diameter, catalogue number 07 625-26) connected the inlet of each microfluidic channel to a multi-channel syringe pump (Model NE-1600, New Era Pump Systems Inc., Wantagh, NY, USA), while Tygon tubing transferred the effluent to a waste container.

The connective tubing and channels were disinfected with a once-through flow of ethanol for 1.5 h, followed by sterile distilled water for a minimum of 24 h. The rinsing water was replaced with sterile growth medium and any air bubbles were flushed out prior to stopping the flow for inoculation. A sterile needle and syringe were used to inject 0.05 mL of the pre-culture containing on average 10^7^ CFU mL^−1^ into each channel, by puncturing a hole through the upstream silicone tubing. The hole was sealed immediately afterwards with all-purpose silicone sealant, and liquid flow was initiated after 10 min at different flow velocities with the syringe pump. The sterile distilled water and growth medium were placed in an ultrasonic cleaner and sonicated at 40 kHz for 20–40 min to remove excess dissolved gases prior to aspirating the liquid into sterile syringes and connecting the syringes to the inlet tubing.

Biofilm development and the associated planktonic cell yield were determined at four different growth medium flow velocities (28.6 × 10^−3^, 29.1 × 10^−2^, 48.5 × 10^−2^, 97.0 × 10^−2^ mL h^−1^) resulting in laminar flow in the channels. The corresponding average shear stresses were calculated by Equation (1) below, and are noted in Table 1 together with Reynolds number for each of the four flow rates.

(1) $$Shear\;stress\;(Pa)=6\times u\times Q/w\times h^{2}$$

where *u* is the dynamic viscosity (taken as 0.001002 Pa.s for water at 20 °C), *Q* is the volumetric flow rate (m^3^ s^−1^), *w* is the width of the channel (m) and *h* is the height of the channel (m).

### 3.4. Biofilm-Derived Planktonic Cell Yield

#### 3.4.1. Viable Cell Counts

The number of viable cells that were released from the biofilm and became entrained in the bulk-liquid phase was determined using serial dilution in sterile saline (0.9% NaCl) and drop plating onto agar-solidified-modified AB medium, containing 10 mmol L^−1^ citrate. Connectors were emplaced near the outlet side of each channel from which effluent was collected at 24-h intervals after inoculation. The number of colony forming units per milliliter (CFU mL^−1^) of effluent was enumerated after 4 to 6 days of incubation at room temperature and normalized with respect to the growth medium flow velocity (mL h^−1^) and the total internal surface area of the channel consisting of both PDMS and glass (cm^2^) to compare the rate of cell yield (CFU cm^−2^ h^−1^) among biofilms subjected different shear rates.

#### 3.4.2. Qualitative Assessment of Biomass Size Distribution

In addition to daily sampling the effluent for viable cell counts, additional effluent was collected periodically. These samples were incubated with formaldehyde (final concentration 3.8% *v/v*) to preserve cell integrity and prevent growth and cell division, at 4 °C prior to dilution of a subsample (if necessary) and incubation with the fluorescent nucleic acid stain 4′,6-diamidino-2-phenylindole (DAPI) at a final concentration of 20 μg mL^−1^ for 20 min in the absence of light. Each sample was filtered onto a black polycarbonate filter (0.2 μm pore-size, 25 mm diameter, Nucleopore, Whatman), followed by placing the filter on a microscope slide with drop of Citifluor antifade mounting medium (AF2, Electron Microscopy Sciences, Hatfield, PA, USA) and a coverslip. Several microscope fields were investigated at random from each filter (60× oil immersion objective, Nikon 90i epifluorescent microscope, Mississauga, ON, Canada) to qualitatively evaluate the size distribution of detached biomass (single cells *vs*. large biomass aggregates).

### 3.5. Confocal Scanning Laser Microscopy and COMSTAT Image Analysis

The extent of biofilm development in duplicate microfluidic channels was examined at 24 hour-intervals with confocal scanning laser microscopy (CLSM, Nikon Eclipse 90i, Mississauga, ON, Canada), using a 40×/0.75 Plan-Fluor objective with excitation of the green fluorescent protein at 488 nm and detection of emission with a band pass 515/30 filter. Ten microscope fields, each with an area of 101,761 μm^2^ (318 μm × 318 μm), were chosen at random along a central transect starting from the channel inlet, and a stack of images was captured in the *z*-direction at 0.60 μm intervals and stored for subsequent analysis with COMSTAT [33].

A selected number of the COMSTAT functions were used for the analysis of the biofilm: the biovolume of each image stack (μm^3^ μm^−2^), the mean thickness of the biofilm (μm) and the biofilm surface area-to-volume ratio (μm^2^ μm^−3^). The biofilm surface area (um^2^) (*i.e*., the area of the biofilm exposed to the bulk-liquid) was calculated using the results of COMSTAT analysis, by multiplying the biofilm biovolume (μm^2^ μm^−3^) by the *xy* attachment area (101,761 μm^2^), and the surface area-to-biovolume ratio (μm^2^ μm^−3^) for each Z-stack of images. The corresponding values obtained correlated well with the degree of biofilm development, as seen in the extent of substratum coverage and average biofilm thickness. The values were primarily utilized for qualitative purposes to identify potential relationships between the planktonic cell yield from biofilms and the different shear forces that they are subjected to.

### 3.6. Statistical Analysis

The extent of biofilm-derived planktonic cell yield for each of the four growth medium flow velocities was evaluated in 2 independent experimental rounds. Each round consisted of biofilm development in duplicate microfluidic channels. The planktonic cell yield data was analyzed using two-factor Analysis of variance (ANOVA, with replication) at a significance level of 0.05, followed by pair-wise comparisons with Tukey’s *Post Hoc* test.

### 3.7. Comsol Simulations

Shear stress simulations were conducted using Comsol MultiPhysics (version 3.5) on a PC system featuring an Intel Core i5-2400 (3.10 GHz) processor running Windows 7 with 64-bit precision. Course, physics-controlled mesh was verified to produce rapid results without sacrificing accuracy. A segment of the channel was created with dimensions 300 μm × 130 μm with channel length between the inlet and measurement point being chosen as 1 mm. Flow was directed along the axis connecting the inlet and outlet with the inlet velocity calculated based on the flow rate and the channel dimensions. The wall shear stress was measured along the long edge of the channel cross-section (300 μm) for each flow rate and a smoothing algorithm was used to remove small digitization effects related from the course mesh.

## 4. Conclusions

The role of biofilms as a microbial survival strategy is often recognized, such as the continued survival of EPS-encased microbes despite multiple antimicrobial challenges. This study demonstrates that in addition to the prevention of cell washout at high liquid shear rates, biofilms also fulfill a proliferation function with continuous cell release into the bulk-liquid, and that shear rates have relatively little impact on such biofilm-to-planktonic cell yield. Instead, biofilms utilize shear to maintain optimum thickness and metabolic activity, which differs from the traditional view that biofilms primarily consist of slow-growing cells.

As a complement to the conventional conceptual biofilm models, we propose the existence of a biofilm surface-associated zone of planktonic cell proliferation. The results suggest the existence of a zone at the biofilm-liquid interface with high metabolic activity where cells are neither irreversibly attached nor imbedded in the EPS matrix, and where shear rates are too low to cause erosion. Such a surface-associated zone with non-attached cells greatly extends biofilm function and probably consists mostly of cells that do not have a typical biofilm phenotype. This phenomenon emphasizes the need to develop improved techniques to better define the EPS boundaries within which cells are immobilized. It appears possible that the EPS in this region is highly responsive to the prevailing environmental conditions, with microbes having the ability to modify EPS rigidity in order to restrict cell movement and thereby maintaining biofilm biomass under conditions that do not support active metabolism and cell replication, or under conditions conducive to microbial activity to allow cells to move away in order to maintain optimum gradients of nutrients and metabolites.

The production and release of significant numbers of planktonic cells by biofilms has long been disregarded. However, results indicate that the traditional perception of biofilms as promoting microbial survival should be expanded to include the potential to contribute to microbial proliferation.

## Figures and Tables

**Table 1:** Laminar flow velocities in the microfluidic channels with corresponding Reynolds numbers and average shear rates applied to biofilms.

Regime	(i)	(ii)	(iii)	(iv)
Flow velocity (mL h^−1^)	28.6 × 10^−3^	29.1 × 10^−2^	48.5 × 10^−2^	97.0 × 10^−2^
Average shear stress (mPa)	9.42	95.9	160	320
Reynolds number	3.68 × 10^−2^	3.75 × 10^−1^	6.24 × 10^−1^	1.25
